# Integrating community health workers to sustain malaria services in the Greater Mekong Subregion: Findings from implementer case studies

**DOI:** 10.1371/journal.pgph.0004528

**Published:** 2025-05-02

**Authors:** Laura Buback, Kyle Daniels, Tiese Etim-Inyang, Monnaphat Jongdeepaisal, Massaya Sirimatayanant, Panarasri Khonputsa, Naomi Beyeler, Richard J. Maude

**Affiliations:** 1 Institute for Global Health Sciences, University of California, San Francisco, California, United States of America; 2 Department of Global Health, University of Washington, Seattle, Washington, United States of America; 3 Mahidol-Oxford Tropical Medicine Research Unit, Faculty of Tropical Medicine, Mahidol University, Bangkok, Thailand; 4 Centre for Tropical Medicine and Global Health, Nuffield Department of Medicine, University of Oxford, Oxford, United Kingdom; 5 Harvard TH Chan School of Public Health, Harvard University, Boston, Massachusetts, United States of America; 6 The Open University, Milton Keynes, United Kingdom; PLOS: Public Library of Science, UNITED STATES OF AMERICA

## Abstract

Many countries in the Asia Pacific rely on community health workers (CHWs) to care for various health needs. In the Greater Mekong Subregion (GMS), malaria CHWs have been an essential component of malaria elimination. Yet as the malaria burden declines, the role of malaria CHWs in local health systems and communities is changing. There is a need to expand malaria CHW roles to take on the provision of health services beyond malaria. This study sought to understand the process and experience of this role expansion including implementation, financing, policy, and sustainability within the Asia Pacific region. We documented malaria CHW programs that included health services in addition to malaria. We conducted 21 key-stakeholder interviews from thirteen programs in eight countries throughout the Asia Pacific region virtually in English and findings were analyzed using rapid-matrix analysis. Participants were recruited by an online landscaping survey, with an inclusion criterion of five + years’ work experience and English speaking. Governments ran five of the thirteen programs; six were international non-governmental organizations (INGOs), and two were academic. Senior staff from programs that have expanded roles of malaria CHWs or integrated CHW programs explained expansion processes, challenges, and opportunities. We found that integration can occur in multiple program domains and does not necessarily occur in all domains simultaneously. We identified entry points for role expansion: integrated policy and financing, planning, assessments, and research. Operational entry points included the selection, training, motivation, management, supervision, and monitoring of CHWs. Enabling factors included decentralized management structures, health system linkages, commodity provision and referral procedures, and community engagement. While there is not a linear or unique path towards integration, we provide considerations for the policy level, practical implementation steps, and enabling factors for countries in the GMS to consider as they move towards sustainable, integrated malaria CHWs.

## Introduction

Many countries throughout the Asia Pacific region rely on community health workers (CHWs) to provide care for health needs ranging from HIV, tuberculosis, and malaria, to vaccination, maternal and child health (MCH), and primary care. As health priorities have shifted and evolved, these CHW programs have likewise shifted in response. This includes changing or expanding CHW scope for different communicable or noncommunicable diseases, addressing emerging health emergencies, such as COVID-19, as well as achieving Sustainable Development Goal #3 [1,2] for reaching universal health coverage (UHC) [3].

The Greater Mekong Subregion (GMS) is one of the areas where CHWs have been pivotal in the fight against malaria. Trained specifically for malaria-related services, these malaria CHWs have contributed significantly to malaria elimination efforts through community-level testing, prevention, treatment, and education [4,5]. Their work has been instrumental in reducing the malaria burden across the region, where a 77% reduction in malaria cases and a 97% reduction in malaria deaths were achieved between 2012 and 2022 [6]. However, despite these gains, recent resurgences in malaria cases in countries such as Thailand and Myanmar highlight the ongoing challenges to malaria elimination and the need to sustain and adapt these programs [5]. Furthermore, as countries near malaria elimination and the malaria burden declines, the uptake of malaria CHW services, and in turn their role in local health systems and communities, is also changing [7]. Sustaining and maintaining the uptake of malaria CHW services as malaria declines is vital for maintaining continued access to malaria services and expanding access to care more broadly [8].

The nature of CHW work varies across the Asia-Pacific region. CHWs often operate in remote, underserved areas, addressing health inequities by extending access to essential services. Expanding the roles of malaria CHWs to include additional services carries risks and benefits. For instance, while role expansion could strengthen the integration of services and sustain malaria programs and health system resilience, it may also lead to overburdening CHWs or diminishing the quality of malaria-specific care. Previous studies have emphasized the importance of appropriate training, supervision, and incentives to mitigate these risks [8,9].

Despite the growing literature on the importance of integrating CHWs into the formal health system [10,11] and extensive best practices on CHW programs globally [12,13], little is known on this role expansion process, especially for active CHW programs in the GMS. Furthermore, there remains limited formal evidence or guidelines on how transitioning vertical CHW programs to integrated service delivery models, specifically in a malaria elimination context. One study in Lao PDR [8] sought to document and elucidate country approaches in the GMS to CHW integration thus far both to help facilitate country to country learning as well as the formulation of regional and global guidance. There is an opportunity to capitalize on CHW investments made to date to understand better how countries can operationalize the expansion of existing cadres for purposes more conducive to UHC [11]. Given the investments made in these cadres, it is a lost opportunity not to maintain these respected workforces to contribute to evolving public health priorities, whether malaria, UHC or the next evolving pathogen [14].

In the GMS, there is growing interest in two strategies for role expansion: (1) enabling malaria CHWs to take on broader health services beyond malaria and (2) extending CHWs who currently focus on other diseases to include malaria responsibilities. Regional strategies emphasize that such integration could provide a sustainable approach to maintaining malaria elimination efforts while improving overall access to healthcare at the community level [15,16].

This study aimed to document CHW programs that included health services in addition to malaria in the Asia Pacific region. Specifically, we explored the process and experience of malaria CHW role expansion, including implementation and management of expanded services, training, monitoring, supervision, financial costs, funding mechanisms, and policy considerations affecting sustainability. By building evidence on the integration of expanded CHW roles within the context of malaria elimination in the Asia Pacific region, this study aims to inform strategies for enhancing community-level healthcare delivery in the GMS.

## Materials and methods

This qualitative study aimed to understand the perspectives of crucial implementing program partners on the experiences and considerations for implementing expanded malaria CHW programs.

### Study design and context

This study was one of a series of studies designed to build evidence on effective strategies for expanding the roles of malaria CHW in the Asia Pacific region, the results of which are presented in accompanying papers in this series. This included a systematic review of CHW programs providing services in addition to malaria in the Asia Pacific [17], a landscaping survey of national malaria programs and implementing organizations in the Asia Pacific [18], and implementation research at the country level. This study was designed to complement the findings of those studies and capture representative examples of the diverse range of Malaria CHW programs. Research questions were developed to target program leaders to understand more details of findings reported in the landscaping survey.

### Participants

**Fig 1:**
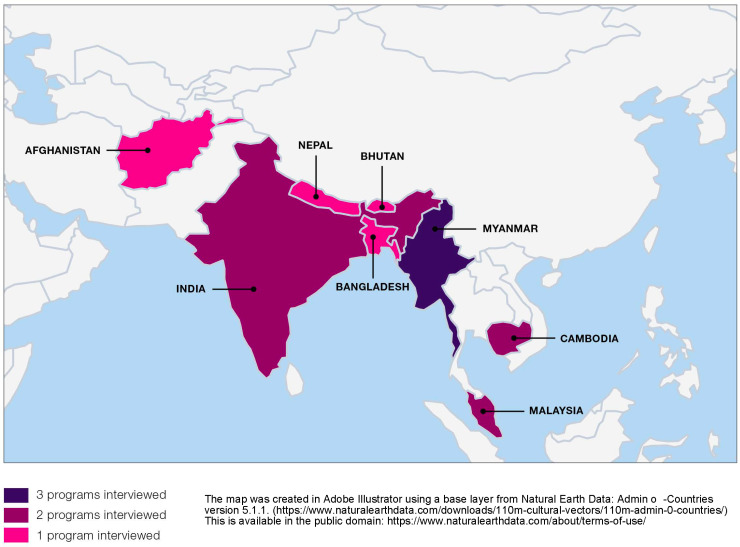
Map of countries represented in interviews of malaria community health worker programs in the Greater Mekong Subregion, November 2021 to February 2022.

The study included interviews with thirteen governmental and non-governmental programs in eight countries throughout the Asia Pacific region, as shown in Fig 1. 21 interviews were conducted from 1^st^ November 2021–11^th^ February 2022. Participating programs were identified through the systematic review [17] and landscaping survey [18] conducted with Ministries of Health and Asia Pacific Malaria Elimination Network (APMEN) country partners, namely national malaria control programs and implementing partner organizations. The landscaping survey targeted staff managing or implementing CHW programs providing malaria services in Asia-Pacific countries. Survey respondents nominated program representatives to be interviewed based on expertise. Inclusion Criteria was: representatives of programs that are currently active and have experience expanding the role of CHWs beyond malaria were contacted for an interview; manager had at least five years of experience in the malaria or community health field; English speaking. Participants’ titles varied by program and country, including program managers, technical officers, operations staff, and country directors. Participants included 15 men and six women; and seven from government and 14 from non-governmental organizations.

### Ethical considerations/consent

This study was approved as exempt by University of California San Francisco Human Research Protection Program Institutional Review Board (UCSF IRB # 21–34766; reference no. 321480). Participants were contacted by email to request participation and obtain consent. All respondents were provided with an information sheet and a consent agreement form. Respondents provided written informed consent by emailing the consent agreement to the study team.

### Data collection

The trained study staff of three interviewers conducted audio-recorded virtual interviews in English. Interviews were conducted virtually by Zoom. Interviews lasted 45–60 minutes and were conducted by at least two study staff, including one interviewer and at least one note-taker. Interviews used a semi-structured interview guide covering the domains of inquiry, which included: human resources, logistics, community engagement, financing, policy, sustainability, success factors and challenges. The interview guide was developed by the study team to align with research questions and pre-tested with two respondents, then revised, before implementation. The complete guide can be seen in S1 Appendix. Notes were reviewed and verified by the interviewer after each interview, including notes on potential biases or challenges for each interview. Recordings were reviewed to verify when responses were unclear or direct quotes were noted. Recordings were not directly transcribed.

### Data analysis

After data collection, all findings were transferred into an analysis matrix organized by a priori key themes. These key themes were identified based on the research questions and existing literature on CHWs. The themes were analyzed iteratively to develop the fundamental entry points. Entry points were identified by triangulating the themes from stakeholder interviews with core components within the literature on sustaining CHW programs, including alignment with the WHO Evidence Guide for Health Policy and System Support for CHWs [12]. Rapid matrix analysis [19,20] was used to organize all interview responses, first categorizing all participant responses in one matrix in Microsoft Excel, then synthesizing all responses within the matrix manually. This rapid qualitative method has proven to be useful in resource-constrained contexts to promote timely uptake of findings by implementers [21,22] and was most applicable to our resource-constrained settings.

While analyzing findings, the following definitions were used for horizontal and vertical approaches to health service delivery [23]: *Horizontal delivery* of health services are is through public financed health systems and is commonly referred to as comprehensive primary care [24] whereas *vertical delivery* of health services implies a selective targeting of specific interventions not fully integrated in health systems [25,26].

## Results

To assess the integration process, we analyzed each program case along two dimensions: First, we assessed six “entry points” for integration, which represent core domains of program design and implementation. Second, we assessed the extent to which each program was integrated along each entry point and the challenges it faced in integration.

The spectrum is plotted across six entry points to expand malaria CHW roles that were discussed by participants. These opportunities included policy, financing, planning, and research considerations. This also included operational entry points for practical steps of selection, training, and motivation, as well as management, supervision, and monitoring of CHWs. Additionally, enabling factors for integration included health system structures and linkages (decentralization, supply chains, and referral mechanisms) and community engagement and strategies for hard-to-reach areas.

Thirteen programs were interviewed from eight countries, represented by 21 respondents. The programs included five government/Ministry of Health (MOH) programs, six international non-governmental organization (INGO) programs, and two academic programs. Respondents were generally program managers and technical advisors of programs that have expanded roles of malaria CHW or integrated CHW programs. Some INGOs also had experience providing program management or technical assistance support to multiple country programs. Table 1 lists the distribution of program types within the different countries and shows a visual representation of the spectrum of integration reported for each country.

**Table 1:** Spectrum of integration within program case studies.

Country	Program Type	Policy & Planning	Finance	Health System Linkages: Referral & Commodity Management	Management/ Supervision of CHWs	CHW Training	Community Engagement
Afghanistan	Former government						
Bangladesh	INGO						
Bhutan	Government						
Cambodia	INGO						
India	Government & INGO						
Nepal	Government						
Malaysia	Government						
Myanmar	INGO/Academic						
(1) Not integrated, malaria/single disease focused							
(2) Somewhat integrated							
(3) More integrated							
(4) Fully integrated primary health care approach							

Among the case study programs, some were highly integrated (integrated across most programmatic domains) while others were largely vertical, with small elements integrated. Table 1 represents this variation, with darker green representing the most integrated, or “horizontal” approach, and lighter green representing the most vertical approach. The scale was based on qualitative perspectives, perceptions, and opinions of stakeholders interviewed about their respective programs and operating contexts.

Overall, the participating programs had the highest level of integration in policy and community engagement. In policy, this reflects the focus on universal primary healthcare and the advocacy and international attention towards policies that embrace a horizontal, primary healthcare-focused approach, such as development of national community health policies. In community engagement, this reflects that stakeholders less often distinguish different donor or national programs. Rather, participants reported examples of community stakeholder bringing together different health concerns in the same forums and utilize the same volunteers, regardless of payment or management structures. There was a much lower degree of integration in finance, management, supply chain, and training – in other words, the operational components of CHW programs. This represents the lengthy process of implementing policy at the operational level due to the challenges of securing financing for policy implementation, sharing financing across sectors, and coordinating actors.

**Fig 2:**
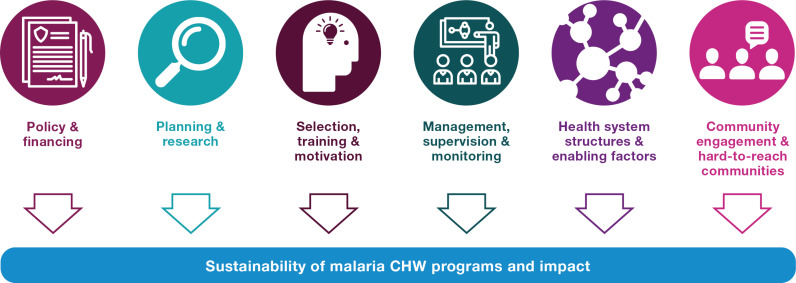
Six entry points to expand malaria CHW/CHW roles and sustainability within community health services in the Asia Pacific region. The images used in this Figure are from https://thenounproject.com/. Terms of Service: https://thenounproject.com/legal/.

Across studied programs, integration began within different program dimensions or health system levels. For example, some programs began the integration process by launching a national level working group to develop policy guidelines for expanded CHW cadres. In contrast, others approached integration from a programmatic level, such as by re-developing the content of community health committee meetings to cover additional diseases or explore emerging health concerns. As shown in Fig 2, leveraging some or all these entry points contributes to increased sustainability and impact of malaria CHW and CHW programs. Participants described both promising approaches and challenges related to each entry point. Case studies for each entry point are listed in Table 2 and approaches and challenges are further detailed in S2 Appendix but synthesized below.

**Table 2:** Entry points & promising case studies.

Entry Point	Case Studies
**Policy & Financing**	Both Bhutan and Malaysia include CHW programs in the malaria and primary health care sections of the national health budget. In India, the Malaria Elimination Demonstration Project (MEDP), piloted an innovative public-private partnership model. In Bangladesh, BRAC is piloting an “Enterprise Model” in which CHWs sell family planning commodities to make a small livelihood, a model that could additionally be adopted for malaria diagnosis and treatment. Countries with integrated, government-run CHW programs, such as Afghanistan, Bhutan, and India, have strong national primary health care package policies, offering integrated services through community-based volunteers/workers funded and managed by national institutions.
**Planning & Research**	In Myanmar, there is an example of soliciting qualitative community perspectives on health needs that was transformed into a pilot on integrated CHW roles. The Royal Government of Cambodia (RGC) conducted mapping exercises in 2017, aiming to identify village health volunteers, map the degree of overlap in HIV, TB, and malaria programs, develop a volunteer database, and utilize results of mapping for the longer-term purpose of integrating roles and responsibilities at the community level.
**Selection, Training & Motivation**	Bangladesh developed and distributed flipcharts to malaria CHWs that included images on TB, vaccination, and other health messages to support sustainable training for malaria CHWs to put new health education messages into practice in community conversations. In Malaysia, the government provides non-financial incentives such as certificates, an ID card, uniforms, training, and most notably, CHWs are offered free treatment at their local health facilities, upon presentation of their badge. In resources scarce settings, remuneration may vary according to responsibility level: In Nepal, “village malaria workers” receive a monthly compensation, while “female community health volunteers” – who support surveillance activities but do not conduct malaria testing and treatment – received a transportation allowance only.
**Supervision, Management & Monitoring:**	In Myanmar, community mobilizers act as village level supervisors of Integrated Community Malaria Volunteers (ICMVs), working with ICMVs to collect hard copies of reports, replenish commodities, provide motivation and technical support to ICMVs, talk and understand their needs, and assess their level. Between visits, Community Mobilizers also keep in touch remotely through phone & monthly meetings with groups of 5–15 ICMVs. The advance tour plan by MEDP India not only provided a tool for malaria CHWs to guide their daily work schedules, but also is akin to quality improvement as it facilitated community perception of malaria CHW/CHWs as “reliable” and “efficient.” Bhutan provides an example of community data use as a component that both engages community and motivates and retains the community workforce. Community level monitoring led by malaria CHWs tracking malaria cases in relation to targets was critical for both the community and malaria CHWs to see results of their work and progress towards malaria free.
**Health system structures & enabling factors**	In Malaysia, medical health officers at the district level have played an instrumental role in expanding the roles of malaria CHWs, as the district level’s flexibility was essential to mobilizing existing volunteers for COVID vaccinations. Decentralized structures: The local and provincial steering committees in Nepal facilitate nimble CHW roles, and the Mitanin CHW model in Chhattisgarh state, India, provides an example of adapting a national program to the context of the state’s unique needs. Some CHWs were provided Rapid Diagnostic Tests and medicines by their state government, whereas other programs only had supplies available to them as part of promotional activities. To replenish commodities, some CHWs come to a central office once a month to stock up on supplies as needed to address and/or prevent outbreaks.
**Community Engagement & hard to reach communities**	CHW/malaria CHWs have improved acceptance and respect when also engaged and embedded within community health committees (CHCs) that facilitate community meetings or conversations, such as Family Health Action Groups (FHAGs) in Afghanistan, Ward Committees and Community Scorecards (CSC) in Bangladesh, and Community Action Groups (CAGs) in Bhutan. Mobile malaria posts in Myanmar and mobile health camps in Bangladesh provide mobile outreach and can expand to include other content outside of malaria. In Afghanistan CHWs provide maternal and child health services in their home villages through family health houses to address shortage of health care providers in remote areas. In places with this infrastructure, malaria testing and treatment services can be integrated into this.

### Policy level entry points: Financing, planning and research

Policy and financing are linked and provide an opportunity to strategically shape the roles of malaria CHWs in national healthcare priorities. Yet, the challenges reported around national community health and primary healthcare policies include a lack of coordination, funding limitations, and varying contexts that require different approaches. Moreover, there is a lack of consensus on appropriate scopes of CHW (i.e., diagnostics, treatment) in the Asia Pacific region, demonstrated by our varied participants. To address these challenges, policy enablers of integration such as strong coordination across national programs, integrated funding, evidence-based guidelines, and decentralized management structures are being implemented. Well-integrated CHW programs are observed in Bhutan, India, and Bangladesh [27–32], which have national solid primary healthcare policies and clear guidelines on the responsibilities and training of CHWs. One INGO participant explained the importance of joint planning and decentralized management to implementation success of an expanded CHW program:

*“An optimal model must satisfy the demand from the community and ministry and stakeholders to be sustainable…Ownership and sense of belonging is very important…we transferred the ownership of the project to the lower level and closely collaborated with staff from regional and state health departments. Regional public health department staff did the data collection, training, monitoring and supervision. In this way, we jointly implemented the project.” (5.C)*

Most participants reported the malaria CHW programs in their countries have primarily been donor funded, rather than domestically funded, for operation and management. Thus, without a clear plan to financially support the full implementation of integrated policies, the sustainability of these programs is uncertain. To address this issue, countries like Bhutan and Malaysia have included CHW programs in their national health budget. Other countries are experimenting with innovative public-private partnership financing models to scale integrated malaria CHW programs, such as the Malaria Elimination Demonstration Project (MEDP) in India [27,28]. An NGO Program Manager from Bangladesh, explained that NGOs support more than half of the CHWs in Bangladesh and one NGO recently provided startup supplies for the “enterprise model”. In this model, CHWs sell family planning commodities to make a small livelihood which showed CHWs can be accepted to sell products. It was reported that “the community already knows TB and malaria services are free…so the community may not be receptive if they sell those” (B.2), this model may be applicable for malaria diagnosis and treatment in other contexts.

The planning landscape of national MOH departments/programs plays a crucial role in integrating disease management and expanding malaria CHW roles, but siloed structures within the MOH were reported to hinder integration. Departments and units tend to prioritize expanding services for the health areas under their purview, rather than identifying opportunities for expanding scope to new health areas. In countries with extensive implementing partner (IP) support, MOHs will likely need to play a more active role in directing planning processes and allocating resources across national health goals. Only in limited circumstances did communities have a role in planning for expanded roles, despite the importance of community engagement in generating acceptance and use of integrated services. Approaches such as soliciting community perspectives on health needs, harnessing planning for UHC, and identifying “Champions” [33,34] within MOHs to facilitate joint planning and coordination of program budgets were reported to contribute to stronger integrated programs.

The research and evaluation of malaria CHW programs play a significant role in expanding and integrating their roles in the healthcare system. They also play a critical role in improving program managers’ understanding of the existing human resources and health priorities of communities and CHWs. Approaches such as mapping and landscaping malaria and other CHWs [35], formative research, and participatory research on community needs and CHW perspectives are emerging as best practices to align program design with the needs and context of local communities. Examples include the mapping exercises conducted by the Royal Government of Cambodia [36] and participatory research conducted with CHWs and communities by the Burnett Institute in Myanmar [37,38]. Additionally, post-training assessments and surveys of CHWs have been used to provide insight into community demand and needs, prompting implementing organizations to adapt their plans and materials to meet community needs when undertaking integration planning and implementation.

### Practical entry points: Selection, training, motivation and management

Selection and training are important to ensure staff can provide the necessary set of services, that these services are high quality, and that they are accepted and taken up by the community. Participants emphasized the need for speaking local languages, and understanding the movements and occupations of community members. While CHWs should ideally be selected from and trained within the communities they serve [13], this was reported to be problematic in remote areas where it is more challenging to find local staff. This was attributed to the limited availability of candidates meeting requirements, often necessitating recruitment from neighboring communities or towns, especially as educational criteria increase for expanded roles. To address this, strong community involvement and collaboration with local authorities help identify suitable candidates, with additional education or literacy criteria applied as needed for expanded responsibilities. Certain programs highlighted that some cadres were systematically recruited from mothers’ groups, as in Nepal, or through existing Village/Community Health Committees, as in program in Myanmar.

Funding constraints often contribute to insufficient training duration and frequency. However, programs have implemented a variety of approaches to address these challenges, such as selecting CHWs from neighbouring communities, providing group and one-on-one trainings with clear guidelines and appropriate materials and job aids, and utilizing post-tests and assessments to evaluate readiness for expanded roles. Refresher training is also crucial for CHWs to practice and improve their new and expanded skills, particularly for uncommon health issues.

The issue of motivating and remunerating CHWs for expanded roles and new tasks is complex, with no clear consensus across programs and countries. Some CHWs prioritize duties with higher incentives, and there are concerns about the sufficiency of current incentive schemes to support programs as malaria CHWs expand their work to take on roles beyond providing malaria services. Non-financial incentives, such as transportation, clothing, and health benefits for the CHWs, can be considered. Some programs have increased compensation rates or provided certificates to CHWs to legitimize their roles. In resource-scarce settings, remuneration varies by responsibility level, and timely payments are crucial to sustaining motivation. Some programs reported that expanding health education responsibilities may be easier than expanding diagnosis and treatment responsibilities. Perspectives on incentives also varied depending on program type. One NGO respondent urged that “in their area, most community health workers are still doing a regular job or other activities so they cannot dedicate all their time to this work; Of course it’s not sustainable, people won’t ride their bike all day in the mud without being paid” (3.B). Yet, another participant from a government program had a varying view: “If the volunteers are not active, they will be accessed by other programs…they are volunteers, they like to volunteer” (11.A). These participants demonstrate the lack of consensus on CHW motivation and remuneration.

Management and supervision are essential to complement CHW motivation, but also to ensure that integrated programs maintain high quality as the scope of services expands. The functionality of malaria CHWs relies on a structure of supervision, which is essential for introducing and maintaining new skills and responsibilities. However, there are several challenges to effective supervision and management for integration, including insufficient human resources, accessibility to target areas, and lack of supervision structure. To address these challenges, some programs reported to concentrate supervisory resources on lower performing malaria CHWs, while others use models of integrated training and supervision [39] or weekly “advance tour plans [40]” for monitoring. Additionally, managers cited the importance of providing ongoing support in supervision, training, and management roles to ensure the sustainability of programs and transitions to government management should acknowledge the high volume of INGO/IP staff currently employed for supervisory needs.

The collection and use of data are essential for effective malaria CHW programs, yet many challenges exist. Inconsistent staff and remuneration threaten health facility-level data collection, and delays and data quality issues are expected at the community level. However, some programs use real-time reporting through mobile phones or paper reporting during monthly supervision visits to overcome these challenges. Community data use is also critical, as it engages the community and motivates and retains the community workforce [25]. Approaches such as community-level monitoring led by malaria CHWs tracking malaria cases in relation to targets have been successful in Bhutan and have been modeled in other countries.

### Enabling environment entry points: Health system and community level factors

Decentralized management structures for malaria CHW programs offer flexibility to adapt and expand responsibilities. This was reported by some as a solution to challenges reported at the operational level, such as long timelines for policy updates, heavy reliance on non-governmental or private sector implementing partners, and delays in procurement due to slow national procedures and lack of sub-national capacity. Approaches such as the use of medical health officers in Malaysia to guide the expansion of the roles of volunteers during the COVID-19 pandemic, the nimble CHW roles facilitated by local and provincial steering committees in Nepal, and the Chhattisgarh Mitanin [14] within India’s National Rural Health Mission (NRHM) demonstrate successful adaptation of national programs to unique local contexts.

The success of expanded malaria CHW roles depends on strengthened health system linkages, which can be achieved through improvements in referral systems and commodity supply-chain. However, several challenges need to be addressed, such as transportation challenges that impede access to primary care in rural and remote regions, lack of trust in community level care, insufficient commodity stocks during outbreaks, and vertical supply systems that can threaten timely and efficient stock management [41,42]. One participant explained that “CHWs had complained that they didn’t receive enough supplies to have an impact” (5.E). To address these challenges, reported solutions include improving referral systems and transportation routes, providing CHWs with necessary supplies, accurately forecasting and quantifying commodities, and integrating supply chain infrastructure.

The success of expanding malaria CHW roles and introducing new services or tasks relies on community engagement and support. However, some obstacles were reported challenging CHW acceptability within communities, especially when CHWs were affiliated with nearby health facilities. These included limited availability of health supplies and unfavorable reputations of nearby health facilities, competition with private pharmacies for affordable treatments some areas of the Asia Pacific, and exceeding demands of malaria CHWs’ capabilities. To overcome these obstacles, one promising approach is to embed malaria CHWs in community health committees (CHCs) to improve acceptance and respect, which cover all health concerns, including malaria, such as the Community Action Group (CAG) in Bhutan [29]. These committees can also collaborate with CHWs in community dialogues to understand community health concerns and needs. Community members must also be informed about the services CHWs offer and their role in the healthcare system to build trust and establish effective communication channels. In some cases the CHC also ensures a positive link and reputation to the nearest health facility.

Expanding malaria CHW roles in hard-to-reach and remote communities requires unique strategies. A participant from Nepal noted the impact of remote border areas on elimination:

*“There are 26 borders (Nepalese) bordering with India and these borders are porous and India is facing malaria cases throughout. Community people move to some states of India… then they come back to Nepal with malaria cases. So, if we don’t treat the imported malaria cases timely, then it can be a problem of transmission in the community. We succeeded to limiting indigenous cases to 32, which is “very low”, but the problem is that there are so many imported cases, around 300 imported cases in 2021” (6.A).*

Porous areas with frequent movement face challenges including needing more malaria CHWs to ensure regular outreach and coverage, infrequent supervision visits, and dysfunctional referral systems. Mobile outreach programs and integration of malaria services into existing maternal and child health infrastructure [39], or “family health house”[43,44] have been used in some contexts. Cross-border collaboration is also important for addressing imported cases and transmission, with the WHO Regional Action Plan [9] towards a malaria-free South-East Asia region emphasizing regional initiatives as a guide for malaria CHW role expansion.

## Discussion

This study demonstrates that there is no one linear, or best, path to integrating and expanding malaria CHW roles. Instead, each program can take a unique path, integrating across different entry points within the program to expand services under various operational and program constraints. In this paper, we have given an overview of important considerations for programs and program funders as they think about integration. Given the diverse contexts, policies, health systems and health priorities of countries and communities in the region, there is no uniform starting point for malaria CHW role expansion among participating programs. To understand integration processes, considerations, challenges, and opportunities, we explored how each program used different entry points for integration.

Malaria CHW programs throughout the Asia Pacific region implemented diverse strategies to expand the scope of malaria CHW practice. This included integrating different health services into the malaria CHW scope of practice, expanding or integrating various components of malaria CHW programs (e.g., training, financing, supervision), and bringing together community-based activities across health areas. Throughout the varied programs and operating contexts, we have identified some key considerations for programs to consider as they decide which path to malaria CHW role expansion best suits their program and country context. These considerations are detailed in S3 Appendix but summarized below. While lessons have been gleaned from the Asia Pacific region, the considerations are considering the GMS context specifically.

Coordination of funding and policy requires improvement at the national level and regionally within the GMS. At the national level, this can be achieved when multiple departments and programs within MOHs jointly identify and agree on the roles of various CHW cadres and develop guidelines and policies accordingly identifying opportunities to fund malaria CHW and other CHW costs jointly. This increases the efficiencies of CHW programs and sets national expectations for the expanded role of integrated community-level cadres. Multi-sectoral collaboration within environmental or education ministries may also be considered for harmonizing scale, scope, and services. This is facilitated by identifying and supporting “Champions” of integration. “Champions” of integration are high level stakeholders who maintain momentum to high level change through commitment to partnerships and collaboration across programs/sectors that do not often work together [26,27]. Promoting cross-program collaboration promotes integration, but is a high-level change for many countries. Thus, a respected, committed “Champion” is needed to drive this forward. Furthermore, regional coordination within the GMS on border-specific strategies for malaria CHW integration and scope expansion is needed to provide guidance, support, and coordination for GMS countries going through similar transitions to expanded CHW roles. Many border areas are served by CHWs of both border countries, so it remains important to harmonize roles across borders. Public-private partnerships, or designated partnership with the private sector to mobilize additional resources that can contribute to CHW costs/needs, can also be used for leveraging supplies, incentives, or funding for expanded roles of malaria CHWs can provide sustainable and stronger resources for larger scope.

A concrete opportunity to inform role expansion policy is landscaping of all malaria and other CHW programs to understand the role and function of CHWs, as well as the level of overlap between various cadres operating at community level. This identifies opportunities for integrating and streamlining training, supervision, and supply chain of community health workers. It is also critical to improve the evidence base and conduct further research on volunteers in the GMS context. Mixed methods research with CHWs and community leaders to strengthen and promote evidence-based discussions on the role of malaria CHWs, feasibility and acceptance, and providing antibiotics and other treatments. Participatory workshop methodology holds potential to ensure that the expanded malaria CHW role and package meet the health needs and priorities of the communities served and the volunteers themselves. Further documented evidence can guide policymakers in permitting the expanded ability of malaria CHWs to provide treatment for common or emerging diseases.

At the operational level, the practical steps to consider for implementing essential training and services by malaria CHWs involve cost-sharing, clear guidelines and training materials, expanded incentives, supervision, user-friendly data collection, and data use. Cost-sharing should be explored to finance trainings and mentorship, while standardized materials based on official guidelines should be developed for different cadres. Additional incentives and resources are necessary to support expanded roles, and clear roles and responsibilities for both malaria CHWs and their supervisors must be established that are context appropriate. Streamlined data collection tools and processes should be user-friendly and integrated into existing systems, focusing on data use for action. These considerations are crucial for the successful implementation and sustainability of community demand for services.

Enabling the environment for sustaining expanded malaria CHW programs will include improved referral systems that support a strengthened, expanded malaria CHW role, stable malaria CHW supply chains to ensure adequate supplies, strong community engagement such as through leveraging village/community health committees, and targeted strategies to serve hard-to-reach and mobile populations. Decentralized management structures involve conducting managerial and planning processes at the sub-national level and utilizing local/provincial Steering Committees. This approach offers flexibility and adaptability to address shifting local priorities while facilitating integrated planning at the operational level. A functional referral system is crucial, establishing communication, transportation, and follow-up linkages for malaria CHWs to refer complex cases to nearby health facilities. If malaria CHWs are given expanded roles in diagnosing complicated diseases/conditions, a reliable health structure is necessary for effective referrals. Additionally, a robust supply chain is required to ensure adequate supplies, particularly when expanded roles demand additional commodities. By minimizing the number of vertical supply chains, the consolidation of commodity distribution processes becomes more manageable. Leveraging existing village or community health committees can aid in the uptake and acceptance of new services malaria CHWs provide within communities. These committees already engage in community-based health promotion, tracking activities and health indicators, and conducting various health interventions. Lastly, specific strategies should be developed for hard-to-reach and mobile populations, ensuring they benefit from malaria and other health programs. Leveraging mobile health interventions for malaria can help extend coverage to these populations, while family/maternal health homes can be expanded to encompass malaria and other services. Efficient coordination of supplies, supervision, and outreach is vital in reaching these areas effectively.

### Strengths and limitations

This study complements the other research in this series, documenting representative examples of the diverse range of malaria CHW programs in the Asia Pacific. It reports concrete challenges and solutions as reported directly be program implementers. This study does not offer a comprehensive review of all malaria CHW programs in the region; rather, it provides a snapshot of the spectrum of expanded malaria CHW programs and roles in the region. We also include several opportunities for entry points into expanded malaria CHW roles, based on case examples gathered from the interviews. The domains and categorizations discussed above are based mainly on the perspectives and opinions of respondents, and there may be other examples in each country that show varied approaches. Given the diversity of program, epidemiology, health system, and country contexts, the entry points are not necessarily generalizable or applicable to all countries within the GMS. Still, the considerations should be interpreted within the lens of each country context. We recommend additional formative research at the country level on landscaping existing programs and opportunities for expanded malaria CHW roles, as well as risks and benefits to the local context. Formative research with malaria CHWs is also a critical next step for understanding practical implementation steps, especially surrounding motivation, incentives, and needs.

Perspectives on challenges, implementation, and enabling factors came from individual interviews that may have been subject to social desirability biases or other personal biases especially related to funding, as most respondents’ livelihoods rely on donor funding. The inclusion criteria for English speaking participants only may have also contributed to selection bias. We put in place measures to prevent such biases through clear explanation of objectives, open-ended questions, and procedures for protecting of confidentiality, though such biases may be present.

## Conclusion

CHWs have been effective and continually funded for disease-specific purposes. Still, as malaria epidemiology shifts in the GMS due to elimination efforts, there is a need to reimagine how these cadres function and can best be leveraged. The most successful programs will be flexible and resilient to change, enabling CHW structures to evolve with shifting epidemiology and health priorities, such as during the COVID response and malaria elimination, playing a pivotal role in community resilience and pandemic preparedness as well. We reported on program case studies and possible entry points for malaria CHW role expansion that can be leveraged by countries in the GMS, and beyond, for the continued evolution of CHWs in expanding UHC.

## Supporting information

S1 Appendix Interview guide. (DOCX)

S2 Appendix Annex 1: Challenges and approaches/case studies for each entry point. (DOCX)

S3 Appendix Annex 2: Considerations for future directions. (DOCX)

S1 Checklist Inclusivity in global research. (DOCX)
