# Supplementary material for: Integrating community health workers to sustain malaria services in the Greater Mekong Subregion: Findings from implementer case studies
Source: PLOS Glob Public Health. 2025 May 2;5(5):e0004528. doi: 10.1371/journal.pgph.0004528 (PMC12047754; doi:10.1371/journal.pgph.0004528)
Supplement: S1 Appendix — (DOCX) [file pgph.0004528.s001.docx]

**S1 Appendix.** Interview Guide

Organization Code:

Staff Name Code:

Interviewer Name:

Note-taker Name (s):

Date/Time:

| Hello, thank you for taking the time to meet with us today.  We are conducting operational research to provide recommendations on integration and sustainability of village malaria workers, focusing on the GMS region, focusing on the implementation process of various organizations in the region with this experience. The research is funded by the Global Fund and will be written up in a brief and/or case studies, so we may also seek your input at a later stage in the process. Know that your name will not be shared, though we may use some direct quotes anonymously. We will be taking notes throughout the process. We will ask some open-ended questions but would like this to be an open conversation about your lessons learned.  Thank you for sending the email consent. I just want to remind you that the interview will last about 30-60 minutes. You can skip any questions and also opt out at any time. I want to ask your permission to record.  Do you have any questions before we begin? |
| --- |
| 1. **Staff role & context:** *Can you begin by Introducing yourself and telling us about your role in the program.* |
| 1. **Basics on program operations:** *Can you tell us about the background of your village malaria worker program. Probe for all components*    1. Recruitment/Training    2. Logistics/supplies    3. Data    4. Monitoring/Supervision/Management    5. Community Engagement    6. financing |
| 1. **We really want to know more about the implementation process for introducing this expanded role to your village malaria workers. Can you tell me more about the process for expanding services.**  - *Planning process, i.e decisions of what services to add, community engagement* - *Roll-out process: training, supplies, financing* - *Monitoring: how did you review the process? Have modifications been made to the program?* |
| **2.a. How is the program thinking about scale – is the program scalable, how has scale been approached, and what are the main barriers and opportunities to scaling the program?** |
| 1. **In your opinion, would you consider this expansion a success? Why or why not?**  - *What parts were successful and why?* - *What could have been done better?* - *What would you do differently if doing it again or expanding to other areas, or even nation-wide? What additional* ***support*** *might be needed? Were resources sufficient?* - *Were some services/diseases harder to integrate or have differing community acceptance?* - *How were challenges managed/overcome?* |
| **3.a. What value does the expanded VHW program bring to the wider health system?** |
| 1. **Policy Context: Can you speak a bit about the policy context during the expansion of the VMWs:**  - *How did the policy context shape the program & experience? Opportunities/barriers?* - *What policy considerations are more important for implementation of expanded roles? (i.e. advice for other programs)* |
| 1. **Sustainability:** Lastly we want to reflect on sustainability. Describe the sustainability of the current program.  - *What are the greatest threats to sustainability? How is the program responding to/addressing these threats?* - *What strategies have been implemented to date to strengthen program sustainability?* - *What programmatic, finance, or policy steps should be implemented to sustain the expanded VMW/VHW program?* |
| 1. **What advice would you give to other programs expanding the role of their village malaria workers? What are the key policy and resources they need to be successful?** |
| **Closing:** Thank you so much for your time and honest answers. Do you have any closing thoughts or questions for our team? |
