# Supplementary material for: Integrating community health workers to sustain malaria services in the Greater Mekong Subregion: Findings from implementer case studies
Source: PLOS Glob Public Health. 2025 May 2;5(5):e0004528. doi: 10.1371/journal.pgph.0004528 (PMC12047754; doi:10.1371/journal.pgph.0004528)
Supplement: S2 Appendix — (DOCX) [file pgph.0004528.s002.docx]

**S2 Appendix.** Annex 1: Challenges and approaches/case studies for each entry point

| **Annex 1: Challenges and approaches/case studies for each entry point** | | |
| --- | --- | --- |
| **Entry Point** | **Challenges** | **Approaches/Case Studies** |
| **Policy:** national community health and  primary health care policy documents, strategic plans or elimination strategies. | - The majority of health funding targets specific diseases, leading to policy development that aligns with funding streams and mechanisms and therefore does not foster role expansion and integration. - There is often a lack of coordination and communication from the national to the operational level. - There is a lack of policy consensus in the region regarding the appropriate scope of CHW practice, including malaria CHW/CHW provision of antibiotic treatments. - There are often large variations of context within a country that requires different malaria CHW/CHW approaches to meet the local needs that are in contrast to or unclear within national recommendations. | - Policy enablers of integration included strong coordination across national programs, especially between national malaria programs and other national programs, more integrated funding to incentivize integrated policies, policy and guidelines based on evidence, epidemiological and community concerns, as well as decentralized management structures - In Bhutan, there is a national policy commitment to free healthcare. Through this commitment, the Ministry of Health of the Royal Government of Bhutan has been able to successfully run an integrated CHW program, building on the efficiencies of task shifting to a single frontline provider, and revealing how an integrated CHW can succeed at the community level with full government commitment and funding. - Countries with integrated, government-run CHW programs, such as Afghanistan, Bhutan, and India, have strong national primary health care package policies, offering integrated services through community-based volunteers/workers funded and managed by national institutions. - Bangladesh also recently conducted a consultative inclusive process to develop its integrated CHW Strategy. - National policies that clearly delineate the responsibilities and educational requirements of various cadres of community health workers and that standardize operational and training guidelines |
| **Financing:** The majority of malaria CHW/CHW programs in the Asia Pacific region currently rely on donor, rather than domestic, funding for their operation and management. | - The majority of malaria CHW program and commodity costs are currently financed by the Global Fund and other donors. The overall program costs to operate and maintain a malaria CHW/CHW program are significant, and without a clear sustainability plan, it will be challenging for national malaria programs or ministries of health and finance to absorb these currently donor-funded programs. - Where strong integration policies exist, there is often a lack of funding to support full implementation of these policies. | - Both Bhutan and Malaysia include CHW programs in the malaria and primary health care sections of the national health budget. - Other countries are experimenting with innovative public-private partnership financing models to scale integrated malaria CHW/CHW programs. For instance, in India, the Malaria Elimination Demonstration Project (MEDP), piloted an innovative public-private partnership model. - In Bangladesh, BRAC is piloting an “Enterprise Model” in which CHWs sell family planning commodities to make a small livelihood, a model that could additionally be adopted for malaria diagnosis and treatment. |
| **Planning landscape:** Similar to the policy context, the planning landscape of the national MOH departments/programs is a factor in expanding and integrating malaria CHW/CHW roles, since each department has its own priorities and budget. | - Departments and units within the national or sub-national ministry of health are more likely to expand services for the health areas under their purview, rather than identify opportunities for expanding scope to new health areas. Thus, siloed structures within the ministry of health can hinder integration. - In countries with extensive implementing partner support, ministries of health play a key role in directing planning processes and allocating resources across national health goals such as malaria elimination. Consultation of implementing partners and sub-national actors is sometimes suboptimal and not considered in annual planning to solicit potential areas for integration that have been identified by community and facility level program managers and providers. - Only in limited circumstances did communities have a role in planning for expanded roles, despite strong presence of community health committee platforms and notable importance of community acceptance as an indicator of success. | - In Myanmar, there is an example of soliciting qualitative community perspectives on health needs that was transformed into a pilot on integrated roles. - A few respondents also noted the potential of harnessing planning for UHC to reduce the siloed approach of vertical financing and planning. Identifying “Champions” within MOHs may also facilitate joint planning and coordination of program budgets to contribute to common integrated programs. “Champions” have been used throughout various policy, organizational and behavioral change management processes, such a quality improvement and electronic medical record transformations. Champions are generally influential, respected, and persuasive leaders. |
| **Research and Evaluation:** Assessments before and throughout program implementation are an opportunity to explore and advance planning for expansion of malaria CHW/CHW roles, especially to ensure expanded roles meet the needs of malaria CHW/CHWs themselves and their communities. | - Various levels of leadership (i.e. local, sub-national, or national) do not fully understand or know what CHWs currently exist and what services they provide. Especially for scaling up or integrating national programs, it is difficult to plan next steps without having a clear understanding of the current human resources. - Implementers often lack clear understanding of the health priorities and program needs of communities and malaria CHW/CHWs, especially for larger and diverse areas that may be experiencing changes disease burdens. | - Approaches to map and landscape malaria CHW/CHWs, formative research to plan for malaria CHW/CHW role expansion, and participatory research to engage malaria CHW/CHW and community members in planning processes are emerging best practices to align program design with the needs and context of local communities, such as the Burnett Institute in Myanmar. - For example, the Royal Government of Cambodia (RGC) conducted mapping exercises in 2017, aiming to identify village health volunteers, map the degree of overlap in HIV, TB, and malaria programs, develop a volunteer database, and utilize results of mapping for the longer-term purpose of integrating roles and responsibilities at the community level. - Where formal research and landscaping is infeasible due to lack of finance or other constraints, post-training assessments and surveys of malaria CHW/CHWs also can provide insight into community demand and needs. For example, one implementing partner learned through malaria CHW surveys about malaria CHW demand for treating other health concerns prevalent in their community, thus prompting revised training materials to fulfill these needs. While national guidelines did not concurrently empower volunteers with the ability to treat additional diseases, the implementing organization was able to expand training to include identification disease signs and symptoms. |
| **Selection/Training:** Some countries described their CHW network as nimble and with ample flexibility to absorb new diseases, whereas other countries were concerned about overburdening CHWs with additional tasks. | - While there was a common understanding that CHWs should be selected from their community and with community input, it can also be difficult to find malaria CHW/CHWs meeting requirements in some of the most remote areas, sometimes leading to selection of malaria CHW/CHW from outside of communities they serve. This may increase if educational requirements increase to meet needs of expanded roles. - Some in-service training for new diseases covered in expanded roles was reportedly too short for the content, did not include clear guidelines to be followed by malaria CHW/CHWs, or their supervisors posed challenges. Funding constraints contributed to these insufficient duration and frequency of trainings, as most training require monetary and/or non-monetary incentives and travel allowances for malaria CHW/CHWs and trainers. - Training is particularly challenging for uncommon health issues, as malaria CHW/CHW will have limited opportunity to practice skills learned in training and may require more frequent refresher trainings. | - CHWs are ideally recruited from within the communities they will be trained to provide health services in (malaria-focused or inclusive of other diseases), with a strong link to local authorities such as village/community leaders. These leaders are also an important entry point for facilitate communication of new responsibilities when malaria CHWs expand their role. In some cases, an expanded role may require additional criteria, such as literacy as a minimum requirement. In areas where the community may not be able to find a CHW who meets the designated criteria, local authorities may also identify people from neighboring communities or larger towns to serve the expanded role. - The majority of programs provide introductory, on-the-job, and refresher trainings in group and one-on-one format, and less frequently online trainings. Refresher trainings are an ideal entry point for expanding malaria CHW roles. The duration of pre-service delivery trainings ranged from 3 days to one-week, while the frequency of refresher trainings ranged from monthly to yearly. - With some concern over introducing additional content, effective trainings are dependent on clear and technically sound guidelines for the subject matter, with appropriate job aids and learning materials for the education levels. For many CHWs, this means pictorials and flipcharts, such as those in Bangladesh. - Well-designed post-tests/learning assessments are also crucial in evaluating skill levels to determine CHW readiness to put their new learnings of expanded roles into practice with their communities. |
| **Motivation/remuneration:** There is a lack of consensus across programs and countries on whether taking on additional responsibilities or new tasks warrants additional incentives to maintain motivation of malaria CHWs/CHWs. | - Integrated CHWs have reportedly prioritized duties attached to programs that provide the most incentives. For example, if an MCH program is providing higher incentives than a malaria program, the CHW may put more time towards the MCH activities. - Programs that already struggle to recruit and retain malaria CHW/CHWs of the adequate education level were not confident that current incentive schemes would be sufficient to support the addition of diseases beyond malaria. - There is limited documentation of malaria CHW/ CHW perspectives on expanded roles and the impact on their motivation. | - If financial incentives are not available to sustain expanded roles, countries can consider various non-financial incentives to motivate the expanded roles, such as covered transportation costs, clothing items, and covered health services, such as in Malaysia. - When expanding the malaria CHW/CHW role in Myanmar to ICMV to cover additional diseases, the MOH also increased the monthly compensation rate to compensate for this. However, some perspectives did not find the increase to be sufficient. These incentives are currently funded by Global Fund partners. - Providing certificates to CHWs can help with their social status in the community to further legitimize the role they are playing in the health system and have been provided for completing trainings in additional skills for expanded roles. - In resources scarce settings, remuneration varies depending on responsibility level. In Nepal, “village malaria workers” receive a monthly compensation, while “female community health volunteers” – who support surveillance activities but do not conduct malaria testing and treatment – received a transportation allowance only. This was also the case in Bhutan where “health assistants” received a monthly compensation, but “village health workers” only received incentives to attend trainings. Countries and programs may select only one cadre to expand roles, or expand different cadres to varying degrees while introducing expanding roles. Beginning with expanding health education responsibilities may be an entry point before expanding diagnosis or treatment responsibilities. - If malaria CHW/CHWs are provided financial incentives, it is important that payment mechanisms ensure routine and timely payments, in order to sustain motivation and avoid gaps in service provision. This is especially true if expanded roles will include additional financial incentives. |
| **Supervision and Management:** malaria CHW/CHW functionality relies on a structure of supervision, which provides an entry point for introducing and maintaining new skills and responsibilities. As malaria CHW/CHWs take on new or different responsibilities in expanded program models, supervision is essential for ensuring support and mentorship of new responsibilities. | - Insufficient human resources: Additional roles, new training areas, or new diagnosis and treatment procedures, require financial and human resources to provide ongoing support to malaria CHW/CHWs. This need for regular support in supervision, training and management roles may interfere with the sustainability of programs, especially given the volume of INGO/IP staff currently being employed for supervisory needs. - Accessibility to target areas: Accessibility, transportation, as well as limitations due to COVID-19 in recent years, have posed challenges to regular supervision. - Training alone is not enough to ensure CHWs learn new knowledge and skills. - Lack of structure: CHW/malaria CHWs may have skills and abilities, but do not have direction in how to go about their work on a daily basis. | - Some programs concentrated their supervisory resources on lower performing malaria CHW/CHWs, whereas other INGO programs emphasized their process for collaborating with the government on supervision for community-based diagnoses. - In Myanmar, one partner uses community mobilizers (CMs) as the village level supervisors of Integrated Community Malaria Volunteers (ICMVs), working with ICMVs to collect hard copies of reports, replenish commodities, provide motivation and technical support to ICMVs, talk and understand their needs, and assess their level. Between visits, CMs also keep in touch remotely through phone & monthly meetings with groups of 5-15 ICMVs. - Models of integrated supervision plans from Myanmar and India hold promise. The advance tour plan by MEDP India is akin to quality improvement as it facilitated community perception of malaria CHW/CHWs as “reliable” and “efficient.” |
| **Data Collection and Use:** malaria CHW/CHW programs all require varied forms of data collection, which can be expanded to include monitoring of community health indicators beyond malaria. | - Health facility level data collection is threatened by inconsistent staff and renumeration. A network of malaria CHW/CHW s was seen as a potential workaround for deeper surveillance, especially if facility-based data is not available. - Data quality threatened by challenges with timeliness of data flow and completeness of community level. - Delays in reporting, especially with paper-based systems, are common. - Data use in general, especially at community level, is a weakness, yet has potential for increasing demand for community-based services. | - Some programs try to do real time reporting, such as by utilizing mobile phones, but mountainous terrain, rainy season, insecurity, and network challenges cause delays in data submission. - Other programs described paper reporting being done once a month by malaria CHW/CHWs and submitted to the supervisor during monthly supervision visits. In Afghanistan, each of the health posts has had a monthly activity report (MAR) format used to tally cases for monthly collection by community health supervisors (CHS). Some of these tally sheets are pictorial to meet the CHWs education level. On a monthly basis, the CHS is responsible for collecting the MAR from each health post and combining all the MARs into one report for their coverage area. This consolidated report is then sent to the province and lastly the MoPH. While this data collection system is reliant on health workers receiving renumeration, it demonstrates a methodology that is user-friendly for that specific cadre. - Bhutan provides an example of community data use as a component that both engages community and motivates and retains the community workforce. Community level monitoring led by VHWs tracking malaria cases in relation to targets was critical for both the community and VHWs to see results of their work and progress towards malaria free. Such approaches have been modeled elsewhere, such as using the DHIS2 community level data approach deployed in Bangladesh, and are important for sustainability and introducing new roles of CHWs. For example, if a malaria CHW/CHW begins to diagnose fever, the community can track fevers over time, and this can help sustain respect and value for malaria CHW/CHW roles. |
| **Decentralized management structures:** Several countries operate with decentralized management structures that have enabled flexibility to adapt and expand malaria CHW/CHW responsibilities. | - Long timelines for updating national policy can hinder innovation or the uptake of innovative local practices into policy guidance, and can also be a barrier to programs adapting to rapidly changing local conditions such as the emergence of new health priorities. - Many local level programs rely heavily on non-governmental or private sector implementing partner management. This can impact the capacity of sub-national health systems to take on finance and management of such programs if and when they are integrated into the national or sub-national health system. - Delays in adequate procurement were often attributed to slow and inflexible national procurement procedures and lack of sub-national capacity. | - In Malaysia, medical health officers at the district level have played an instrumental role in expanding the roles of malaria CHWs. An MOH staff explained that these district level staff really know the volunteers, the different cadres, and can make informed decisions about the new work volunteers are able to take on. In Malaysia, there are primary health care volunteers, malaria volunteers, and dengue volunteers, but many of them may serve in multiple roles. This was also the entry point for their involvement in COVID vaccination activities, as the Malaysia team noted the district level’s flexibility was essential to mobilizing existing volunteers for COVID vaccinations. They emphasized the willingness and skills of volunteers needed to be better leveraged by strong leadership. - Likewise, the local and provincial steering committees in Nepal facilitate nimble CHW roles, and the Mitanin CHW model in Chhattisgarh state, India provides an example of adapting a national program to the context of the state’s unique needs. |
| **Health System linkages:**  strengthening the referral structure and commodity supply-chain are important enabling factors for sustaining expanded malaria CHW/CHW roles. | - In many rural and remote regions, accessibility to primary care can be impeded by transportation challenges. Expanded or integrated CHW programs can serve as a critical health service and health system linkage in these underserved areas, and expanding their scope of practice can close critical community health needs. - Lack of supplies and functionality of malaria CHW/CHWs may lead community members to bypass community level care, or public health services in general and opt for private health centers or pharmacies. Furthermore, the community may lack trust in the nearest health facilities and bypasses them for tertiary care. Expanding malaria CHW/CHW services in general should build trust in their functionality and the affiliated systems. - As malaria burden decreases, stocks are generally lower, sometimes resulting in insufficient commodity stocks during outbreaks. Vertical supply systems may be a threat to timely and efficient stock management as routine need lowers but adaptability to outbreaks remain crucial. | - Improving the functionality of referral systems. Programs aimed at reducing maternal and child mortality have also focused strongly on strengthening referral processes and transportation routes and resources, as well as leveraging integration of other health services. - Some CHWs were provided RDTs and medicines by their state government, whereas other programs only had supplies available to them as part of promotional activities. To replenish commodities, some CHWs come to a central office once a month to stock up on supplies as needed to address and/or prevent outbreaks. - The accurate forecasting and quantification of commodities was also raised to ensure supply matches demand and to effectively manage stock for outbreaks and emergencies. Combining or integrating supply chain infrastructure, such as staffing, transportation, and distribution processes may strengthen the ability to provide malaria commodities, as well as others. |
| **Community Engagement:** Introducing new services or tasks to a CHW roles will be more accepted with the support of community leaders and authorities and the community health structures have the best knowledge of community health concerns that are priorities to expand into malaria CHW practice. | - Limited availability of health supplies: When CHWs do not have the necessary supplies or equipment to do their work, it threatens their reputation as reliable service providers, and interferes with their credibility in the community. - Referral to health facilities: Many community members do not want or are not financially able to travel to health facilities. Furthermore, if the nearest health center/health post/health facility has an unfavorable reputation, community members may be less willing to accept referrals from malaria CHWs associated with those facilities. - Pharmacies/private health centers: In areas where CHWs do not provide treatment, private pharmacies can also be a threat to CHWs because they offer medicines and cures, so communities may rely more heavily on pharmacies than CHWs. - Demand exceeds capability: In cases where malaria CHWs are seen as knowledge and valuable, the community may expect or demand more services than the malaria CHW/CHW is trained, equipped, or permitted to manage. | - CHW/malaria CHWs have improved acceptance and respect when also engaged and embedded within community health committees (CHCs). CHCs vary by country but, in many countries, these committees cover all health concerns, including malaria. Examples of such committees include Family Health Action Groups in Afghanistan, Ward Committees and Community Scorecards (CSC) in Bangladesh, and Community Action Groups (CAGs) in Bhutan. As these groups approach health care from an integrated perspective, they are critical to engage as CHW/malaria CHW roles expand to meet shifting needs. - CHCs also often collaborate with CHWs in community dialogues or meetings, which also commonly cover topics beyond malaria and can create a forum for understanding community health concerns, perceptions, and needs. Community members should be informed about the services CHWs offer and their role in the health care system and should be provided opportunities to share their needs from this cadre of health providers. |
| **Hard to Reach/Remote Communities:** Hard to reach, remote areas are both more challenging and more resource intensive to provide community level health services, so may require unique strategies for malaria CHW/CHW roles expansion. It is most efficient to build upon existing malaria CHW/CHWs when expanding services, which may mean expanding the role of malaria CHWs to offer more services, or utilizing existing CHWs to offer additional malaria or other services. | - Given the difficulty and time to travel to some areas, a larger number of malaria CHW/CHW may be required to ensure regular outreach to and coverage of malaria and other health services to households in hard-to-reach areas. - Supervision visits to monitor and restock malaria CHW/CHWs in these areas is often less frequent, providing an opportunity for coordination and consolidating supervision across sectors. - The referral systems as mentioned above are often not functioning to a degree that incentivizes remote populations to utilize services, and may threaten the ability to expand malaria CHW/CHW ability to provide referrals. | - Mobile outreach programs are one example of how countries are working to provide coverage to these areas, such as the mobile malaria posts in Myanmar or mobile health camps in Bangladesh. Such programs can expand to include other content outside of malaria. - In Afghanistan CHWs provide maternal and child health services in their home villages through family health houses to address shortage of health care providers in remote areas. In places with this infrastructure, malaria testing and treatment services can be integrated into this. - To address the issue of imported cases and cross-border transmission, it is important to consider the possible benefit of malaria and other disease program collaboration across borders. The WHO Regional Action Plan towards a malaria-free South-East Asia (SEA) region (2017-2030) includes strengthening of regional cross-border malaria elimination initiatives as one of its aims and calls for the prioritization of collaboration between countries in the SEA region. Additionally, the Regional Civil Society Organization (CSO) Platform was highlighted as an effective starting point for regional collaboration, but other border health initiatives may also be considered. These regional initiatives have an important role in guiding future directions of malaria CHW/CHW roles expansion. |
