# Supplementary material for: Integrating community health workers to sustain malaria services in the Greater Mekong Subregion: Findings from implementer case studies
Source: PLOS Glob Public Health. 2025 May 2;5(5):e0004528. doi: 10.1371/journal.pgph.0004528 (PMC12047754; doi:10.1371/journal.pgph.0004528)
Supplement: S3 Appendix — (DOCX) [file pgph.0004528.s003.docx]

**S3 Appendix.** Annex 2: Considerations for Future Directions

| **Consideration** | **Description** | **Importance for expanded roles** | **Relevant Case Studies** |
| --- | --- | --- | --- |
| ***Policy Considerations*** | | | |
| Increased coordination of funding mechanisms and programs | Multiple departments and programs within Ministries of Health jointly identify and agree on the roles for various CHW cadres, including malaria CHWs, and develop guidelines and policies accordingly identifying opportunities to jointly fund malaria CHW/CHW costs. | Increases efficiencies of malaria CHW/CHW programs and sets national expectations for expanded role of integrated community level cadres. Multi-sectoral collaboration within environmental or education ministries may also be considered for harmonizing scale, scope, and services. | Bangladesh CHW policy coordination |
| Identifying and supporting “Champions” | “Champions” of integration are high level stakeholders who maintain momentum to high level change through commitment to partnerships and collaboration across programs/sectors that do not often work together. | Promoting cross-program collaboration promotes integration, but is a high level change for many countries, thus as a respected, committed Champion is needed to drive this forward. This is ideally at the highest level possible of national policy makers, but may also be at decentralized levels. | No examples yet |
| Regional funding and coordination mechanisms | Regional consensus within the GMS on the role of malaria CHWs together with border-specific strategies for malaria CHW integration and scope expansion. | Provides guidance, support, and coordination for GMS countries going through similar transitions to expanded malaria CHW/CHW roles. Many border areas are served by malaria CHW/CHWs of both border countries, so important to harmonize roles. | Global Fund RAI program & Regional CSO Platform |
| Public-private partnerships | Designated partnership with the private sector to mobilize additional resources that can contribute to malaria CHW/CHW costs/needs. | Leveraging supplies, incentives, or funding for expanded roles of malaria CHW/CHWs can provide sustainable and stronger resources for larger scope. | MEDP Public-Private Partnerships |
| Mapping | Landscape of all malaria CHW/CHW programs to understand the role and function, as well as the level of overlap between various cadres operating at community level. | Identifies opportunities for integrating and streamlining training, supervision, and supply chain of community health workers. | Cambodia CHW mapping |
| Increase the evidence base for volunteers in the GMS context | Mixed methods research with malaria CHW/CHWs and community leaders themselves to improve and promote evidence-based discussions on the role of malaria CHW/CHWs, feasibility and acceptance, and providing antibiotics and other treatments. | Participatory workshop methodology holds potential to ensure that the expanded malaria CHW/CHW role and package meets the health needs and priorities of the communities served, as well as the volunteers themselves. Further documented evidence can guide policy makers in permitting expanded ability of malaria CHW/CHW to provide treatments | Burnett Institute participatory research in Myanmar |
| ***Practical Steps*** | | | |
| Cost-sharing for essential trainings | Training, refresher trainings, and mentorship will be required for all new services, thus financing the movement and gathering of workers, as well as provision of training materials, must be considered. | Training is a key opportunity for cost-sharing and efficiencies across programs or organizations, as CHWs may travel to one venue to receive integrated trainings across multiple subjects, thus reducing costs, though this does require coordination. | Myanmar iCCM refresher trainings |
| Clear Guidelines, Training, and Implementation Materials | Standardized training materials based on official MOH guidelines that are appropriate for the educational requirements of each cadres. For example guidelines and materials for volunteers will need to be tailored for supervisors. | Some expanded programs have struggled in initial rollout due to lack of guidelines and materials to support new or rare diseases/health areas, so it is imperative to have evidence-based guidelines for CHWs to identify, diagnose, refer, or treat additional diseases adopted by the Ministry of Health. | Bangladesh pictorial flip charts |
| Expanded financial and/or non-financial incentives | Additional research and discussion with malaria CHW/CHWs and key stakeholders in each country can provide further insight on the incentives required for additional tasks. | Expanding roles should at minimum include additional certificates and the necessary supplies. Increased opportunities for exchanging with peers and supervisors should also be included as non-financial incentives to facilitate adaptation to new roles. | Non-financial incentives or tiered incentives by cadres |
| Supervision | This may be a higher cadre of community supervisor, midwife, district health officer, NGO staff, or central level MOH. Clear roles and responsibilities for malaria CHW/CHWs and their supervisory cadres must be well documented and well enforced, with adequate resources provided for supervision visits and peer meetings. | it is critical to allocate human and financial resources to intensive supervision of new diseases. | Myanmar and MEDP |
| User friendly, streamlined data collection | Data collection tools and data flow processes in place that have been designed with the end user’s experience, education level, and bandwidth. | Since this is already a common challenge, if expanding the data collected by malaria CHW/CHWs, it must be simplified. Similarly, a strategy for how to integrate the data reporting of CHWs into the surveillance system, and whether necessary to delineate those cases as community identified, warrants consideration. | Afghanistan pictorial data collection |
| Data use | The data collected by malaria CHW/CHWs is regularly reviewed and interpreted for action. | Using data at various levels, especially health center and community, will be critical for sustainability of community demand for services. | Bhutan CAG community data for action |
| ***Enabling Factors*** | | | |
| Decentralized management structures | Managerial and planning processes take place at the sub-national level, such as through local/provincial level Steering Committees. | Provides flexibility and adaptability to shifting local priorities and facilitating planning in integrated roles and functions at the operational level. | Nepal Steering committees |
| Functional referral system | Communication, transportation, and follow up linkages in place for malaria CHW/CHWs to refer complicated cases to nearest health facilities | If expanding malaria CHW/CHW roles to diagnose more complicated diseases/conditions, this includes referrals of complicated cases. This requires a dependable health structure to refer these complicated cases. | N/A |
| Supply chain to ensure adequate supplies | Expanded roles require additional commodities and also decentralized ability to manage stocks will be important if expanded. | Minimizes the number of vertical supply chains that need to be managed and consolidating commodity distribution processes. | N/A |
| Leverage village or community health committees | Existing village health groups conduct community-based health promotion and tracking of activities and health indicators, and many existing health activities. | To ensure uptake and acceptance of new services offered by malaria CHW/CHWs within communities. | Community Action Groups Bhutan / Community dialogues Cambodia |
| Hard-to-reach and mobile population strategies | Specific strategies must be included for these groups who might not benefit from standard malaria or other health program interventions. | Mobile health interventions for malaria can be leveraged to cover other services, and likewise family/maternal health homes focused on maternal and child health can also be leveraged to expand to malaria and other services. Given the resources to reach these areas, it is efficient to coordinate supplies, supervision, outreach. | Family Health Houses in Afghanistan |
